# PD-1 expression on peripheral CD8+ TEM/TEMRA subsets closely correlated with HCV viral load in chronic hepatitis C patients

**DOI:** 10.1186/1743-422X-7-310

**Published:** 2010-11-12

**Authors:** Tao Shen, Jiajia Zheng, Chunhui Xu, Jia Liu, Weidong Zhang, Fengmin Lu, Hui Zhuang

**Affiliations:** 1 Department of Microbiology, Peking University Health Science Center, Beijing 100191, PR China; 2 Department of Epidemiology, College of Public Health, Zhengzhou University, Henan 450052, PR China

## Abstract

**Background:**

Tight correlation between host circulating CD8+ T cell-mediated immune response and control of viral replication is classical characteristic of long-term HCV infection. CD8+ T cell maturation/activation markers are expected to be associated with viral replication and disease progression in chronic HCV infection. The aim of the present study was to explore novel markers on CD8+ T cells with ability to evaluate HCV viral replication and disease progression.

**Methods:**

PBMCs were isolated from 37 chronic HCV-infected patients and 17 healthy controls. Distributed pattern of CD8+ T cells subsets and expression of PD-1, CD38, HLA-DR and CD127 were analyzed by flow cytometry. The correlation between expression of surface markers and HCV viral load or ALT was studied.

**Results:**

Declined naïve and increased TEMRA CD8+ T subsets were found in HCV-infected individuals compared with healthy controls. Percentage and MFI of PD-1, CD38 and HLA-DR on all CD8+ T cell subsets were higher in HCV-infected patients than healthy controls. In contrast, CD127 expression on CD8+ TCM showed an opposite trend as PD-1, CD38 and HLA-DR did. In chronic HCV infection, MFI of PD-1 on CD8+ TEM (p < 0.0001) and TEMRA (p = 0.0015) was positively correlated with HCV viral load while HLA-DR expression on non-naive CD8+ T cell subsets (p < 0.05) was negatively correlated with HCV viral load.

**Conclusion:**

PD-1 level on peripheral CD8+ TEM/TEMRA was highly correlated with HCV viral load in chronic HCV-infected patients, which made PD-1 a novel indicator to evaluate HCV replication and disease progression in chronic hepatitis C patients.

## Background

Hepatitis C virus (HCV) infection was prevalent in several provinces of China due to unsanitary blood collection history in 1990's [1-3]. Traditionally, viral load and alanine aminotransferase/aspartate aminotransfearse (ALT/AST) were used as clinical surrogates to evaluate hepatitis C disease progression and effect of antiviral therapies. Since dysfunctions of viral specific CD8+ T cell immune responses are closely associated with HCV replication [4-6], simple and easy-manipulated CD8+ T cell maturation/activation markers which are able to assess viral replication and/or disease progression are desired.

According to the expression of CD45RA and another co-stimulating molecule CD62L, CCR7 or CD27, CD8+ T cells can be divided into four different subsets: Naïve, TCM (central memory T cell), TEM (effector memory T cell) and TEMRA (CD45RA+ effector memory T cell) [7-12]. Following priming by infected HCV or HIV-1, naïve cells can clonally expand and subsequently differentiate into TEMRA/TEM cells. Once virus was cleared, most activated CD8+ T cells experienced apoptosis and finally a minority of survived effector cells becomes TCM cells[13,14]. It has been proposed that unbalanced distribution of circulating CD8+ T cell subsets and impairment of homing capacity and effector function are closely associated with HCV/HIV-1-specific immune tolerance and viral persistence [15-19].

The PD-1(programmed death-1) molecule is expressed on lymphocytes, especially on T and B cells, and is an inducible inhibitory regulator of T cell activation [20,21]. Interaction between PD-1 and its ligand PD-L1 (programmed death ligand 1), which mainly expressed on antigen-presenting cells (APC) such as dendritic cells (DCs) and macrophages, has been postulated to negatively regulate T cell activation and immune evasion in viral infection and autoimmune diseases[21-23]. Higher level of PD-1 expression in HCV infection was documented [24-26]. Urbani *et al*.[24] demonstrated that PD-1 expression in acute HCV infection seemed to be a signature of functional HCV-specific CD8 T cell exhaustion. Golden-Mason *et al*. [25] and Radziewicz *et al*.[26] showed that PD-1/PD-L1 pathway was critical in persistent of HCV infection and represented a potential novel target for reversible immune dysfunction. Whereas, so far, no defined data were clearly and systematically depicted PD-1 expression on CD8+ T cell subsets in chronically HCV infected patients.

As a subunit (α chain) of IL-7 receptor, CD127/IL-7 system plays an essential role in maintaining homeostasis of circulating T cells, including naïve and memory T cells under physiological as well as immune disordered conditions[27,28]. Though the exact role of CD127 in pathogenesis of HCV and HIV-1 infection is still undetermined, numerous studies showed that CD127 might be a potential predicator of clinical status in both adults and children infected by HCV and/or HIV-1[29-31]. CD38 and HLA-DR molecules were regarded as classic immune activators and were widely used to gating activated T cells in cellular immunology [32-34]. Since HCV infection could induce CD8+ T cell activation and impaired balance of T cell-homeostasis, it will provide more detailed information to assess the status of CD8+ T cell maturation (CD45RA and CD27), activation (CD38 and HLA-DR), inactivation (PD-1) and generation of immune memory (CD127) in chronic HCV-infected patients.

Thus, the aim of the present study was to analyze the status of CD8+ T cell maturation/activation in chronic HCV infected patients in comparison with healthy controls and to explore novel markers on CD8+ T cells with ability to evaluate HCV viral replication and/or disease progression.

## Materials and methods

### Study Participants

54 subjects were recruited and divided into two groups: chronic HCV-infected patients (n = 37), and healthy controls (n = 17). All participants were residents of a village of Henan province and interviewed by trained and qualified staffs using standardized questionnaire. All HCV-infected patients were former blood donors (FBDs) and negative for HBV and HIV infection, which were defined by seronegativity with enzyme immunoassays and Western Blot respectively. HCV infection was confirmed by detectable plasma HCV viral load measured by Abbott RealTime™ HCV Amplification Kit (Des Plaines, IL, USA) and by reactive serum HCV antibodies measured by Abbott ARCHITECT anti-HCV system (Des Plaines, IL, USA). Healthy donors were negative for HCV, HBV and HIV-1 infection. Importantly, none of HCV-infected patients had received any HCV-specific antiviral therapy. The study was approved by institutional review authorities and informed consent forms were signed by all participants.

### Clinical Biochemical Tests

Liver associated enzymes including ALT, AST, γ-GT (γ - glutamyltransferase), ALP (alkaline phosphatase) and other variables such as albumin, globulin and albumin/globulin ratio in serum were measured by clinical standardized methods.

### Flow Cytometry Analysis

Peripheral blood mononuclear cells were isolated from heparinized blood and expression of surface markers on CD8+ T cells were analyzed by BD FACSAria (San Jose, CA) compensated with single fluorochromes and driven by BD FACSDiva software (San Jose, CA). Data analysis was performed using FlowJo (San Carlos, CA). Fluorochrome-labeled monoclonal antibodies specific for CD4-APC-Cy7, CD4-PE-Texas Red, CD8-PE-Cy7, CD45RA-PE-Cy5, CD45RA-APC, CD27-PE, CD27-APC Cy7, CCR7-PE Cy7, CD25-APC, PD-1-FITC, HLA-DR-APC, CD38-FITC were supplied by BD Pharmingen (San Diego, CA), and CD127-Pacific blue and CD8-Pacific Blue were supplied by eBioscience (San Diego, CA). CD3-ECD was purchased from Beckman Coulter (Fullerton, CA).

### Statistical analysis

Data were analyzed with GraphPad Prism 5 software (San Diego, CA, USA). Variables were compared using nonparametric tests (Mann-Whitney U test) and correlation was performed using Spearman rank correlation coefficient test. P values < 0.05 was considered as statistical difference.

## Results

### Characteristics of study populations

Two groups of participants were recruited in this study: chronic HCV-infected patients and healthy controls. The demographic and laboratory characteristics of all participants were summarized in Table 1.

**Table 1:** Demographic and laboratory characteristics of participants enrolled in the study

Characteristics	Healthy control	HCV-infected patients
	(n = 17, F/M = 10/7)	(n = 37, F/M = 21/16)
**Age (years) (mean ± SD)**	42.034 ± 14.321	47.105 ± 16.990
**ALT**^**b **^**level(IU/mL) (mean ± SD)**	n.a.^a^	43.324 ± 26.601
**AST**^**c **^**level(IU/mL) (mean ± SD)**	n.a.	39.250 ± 18.181
**Y - GT^d ^(IU/mL) (mean ± SD)**	n.a.	28.916 ± 25.282
**ALP**^**e **^**level(IU/mL) (mean ± SD)**	n.a.	78.321 ± 36.270
**Albumin level(g/L) (mean ± SD)**	n.a.	47.354 ± 6.966
**Total protein level(g/L) (mean ± SD)**	n.a.	75.461 ± 15.782
**Ratio of albumin to globulin (mean ± SD)**	n.a.	1.410 ± 0.864
**Total bilirubin level(μmol/L) (mean ± SD)**	n.a.	12.280 ± 3.761
**Direct bilirubin level (μmol/L) (mean ± SD)**	n.a.	5.214 ± 5.390
**Hemoglobin(g/L) (mean ± SD)**	n.a.	139.620 ± 14.551
**HCV genotyping (1b/2a)**	n.a.	16/21
**HCV RNA(log**_**10**_**IU/ml) (mean ± SD)**	n.a.	5.747 ± 1.244

^a ^n.a., not available or not applicable; ^b^ALT, Alanine aminotransferase; ^c ^AST, Aspartate aminotransfearse; ^d ^γóGT, γó glutamyltransferase; ^e^ALP, Alkaline phosphatase.

### Impact of HCV infection on distribution of CD8+ T cell subsets

**Figure 1:**
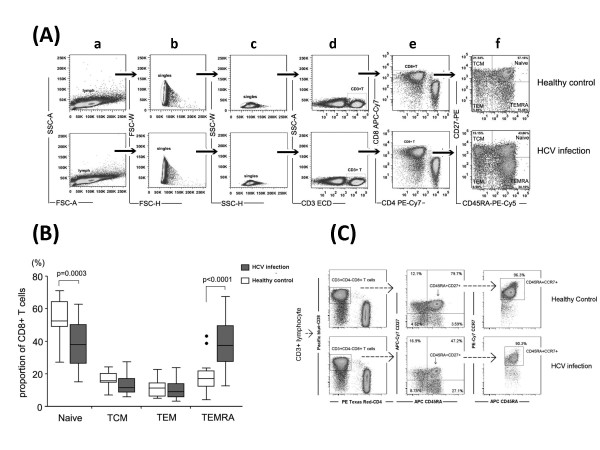
**Distribution of CD8+ T cell subsets in HCV-infected patients and healthy controls according to the expression of CD45RA and CD27**. **(A)**. Representative Dot plots analysis showing the gating strategy to define CD8+ T cell subsets using CD45RA and CD27. The plots from (a) to (f) were first gated on lymphocytes by FSC and SSC, then the CD3+CD4-CD8+ subpopulation was defined by the expression of CD45RA and CD27. **(B)**. Comparison of CD8+ T cell subsets in chronic HCV infection (*dark grey boxes*) and healthy controls (*open boxes*). Data were shown as median and interquartile range values. Symbols: ●, outlier values (more than 1.5 times the interquartile range). **(C)**. Dot plots analysis showing that the majority of CD8+ CD45RA+CD27+ naïve T cells presented CD45RA+CCR7+ phenotype. A representative dot plot result of five HCV patients and five healthy donors,

CD8+ T cells can be divided into four subsets according to CD45RA and CD27 expression: naïve (CD45RA+CD27+), TCM (CD45RA-CD27+), TEM (CD45RA-CD27-) and TEMRA (CD45RA+CD27-). Gating strategy for CD8+ T cell subsets was shown in Figure 1A (a-f). The percentage of naïve CD8+ T cells in HCV infection was significantly decreased compared with healthy controls (p = 0.0003, Figure 1B), while percentage of TEMRA showed an opposite trend, which was increased in HCV infection (p < 0.0001, Figure 1B). Also, as shown in Figure 1C, the majority (more than 90%) of CD45RA+CD27+ CD8+ T cells were corresponding to CD45RA+CCR7+ CD8+ T cells both in HCV chronic-infected patients and healthy controls.

### Expression of maturation/activation markers on total CD8+ T cells in HCV infection

**Figure 2:**
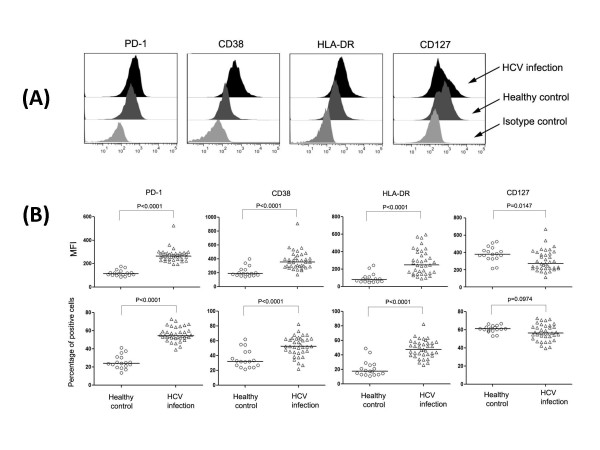
**Comparison of PD-1, CD38, HLA-DR and CD127 expression on total CD8+ T cells in HCV-infected patients**. **(A)**. Representative histogram showing different patterns of PD-1, CD38, HLA-DR and CD127 expression on whole CD8+ T cells in chronic HCV (*dark area*) infection, healthy controls (*dark grey area*) and isotype antibodies controls (*grey area*). **(B)**. Statistic analysis of PD-1, CD38, HLA-DR and CD127 expression on total CD8+ T cells in chronic HCV (△) infection and healthy controls (○). Data were presented as mean fluorescence intensity (MFI) **(top panel) **and percentage of positive cells **(low panel)**. Graphs showed vertical scatter plots and median lines.

PD-1, CD38, HLA-DR and CD127 expression on total CD8+ T cells were measured (Figure 2A) and results were shown as mean fluorescence intensity (MFI) and percentage of positive cells (Figure 2B). Significantly higher PD-1 (p < 0.0001), CD38 (p < 0.0001) and HLA-DR (p < 0.0001) expression were observed in HCV-infected patients (Figure 2A and 2B). Different from PD-1, CD38, and HLA-DR expression, MFI of CD127 was declined in HCV infection (p = 0.0147 < 0.05) compared to healthy controls (Figure 2B). However, no significant difference was found when calculation was performed using percentage of CD127 positive cells (Figure 2B).

### Different patterns of PD-1, CD38, HLA-DR and CD127 expression on CD8+ T cell subsets in HCV infection

**Figure 3:**
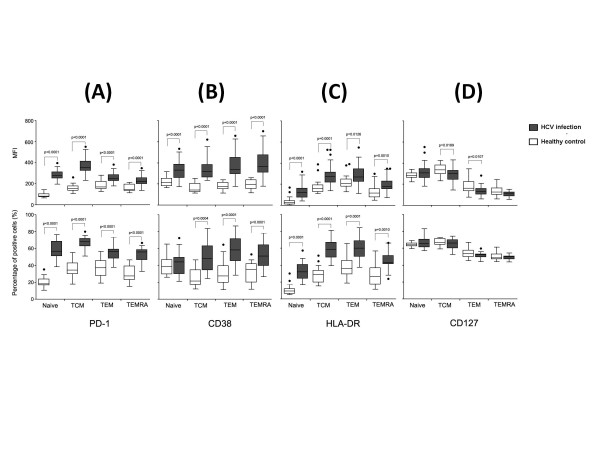
**Comparison of PD-1(A), CD38 (B), HLA-DR(C) and CD127 (D) expression on CD8+ T cell subsets (naïve, TCM, TEM and TEMRA)**. Data were presented as MFI **(top panel) **and percentage of positive cells **(low panel) **between chronic HCV infection (*dark grey boxes*) and healthy controls (*open boxes*). Graphs showed median and interquartile range values of MFI or of percentages of positive cells. Symbols:●, outlier values (more than1.5 times the interquartile range).

The expression patterns of markers (PD-1, CD38, HLA-DR and CD127) on different CD8+ T cell subsets were measured in HCV-infected patients. As shown in Figure 3A, all subsets of CD8+ T cells in HCV-infected patients expressed higher PD-1 in both MFI and percentage (p < 0.0001), compared with healthy controls. The highest MFI of PD-1 or proportion of PD-1 positive cells were found in TCM subset, and to a less degree were naïve cells in HCV-infected patients. Similar to PD-1, HCV-infected patients expressed significantly higher MFI and percentage with regards to expression of CD38 and HLA-DR on most CD8+ T cell subsets (Figure 3B). MFI of CD38 on all CD8+ T cell subsets increased in HCV infection than healthy controls (p < 0.0001). Both MFI and percentage of HLA-DR positive cells were increased on all CD8+ T cell subsets in the HCV-infected patients than healthy controls (Figure 3C, p < 0.0001). We also found that HLA-DR expressed higher on memory CD8+ T cells (TCM/TEM) than effector subsets (TEMRA), indicating HLA-DR expression would drop when CD8+ T cells developed from memory to effector status, though both memory and effector CD8+ T cell subsets expressed significantly higher HLA-DR than naïve CD8+ T cells. As shown in Figure 3D, MFI of CD127 was declined on TCM and TEM in HCV-infected subjects (p < 0.05) compared with healthy controls, while no similar trend was found when CD127 expression was presented as percentage of positive cells.

### Correlation analysis of maturation/activation markers on CD8+ T cell subsets with traditional clinical indicators

**Figure 4:**
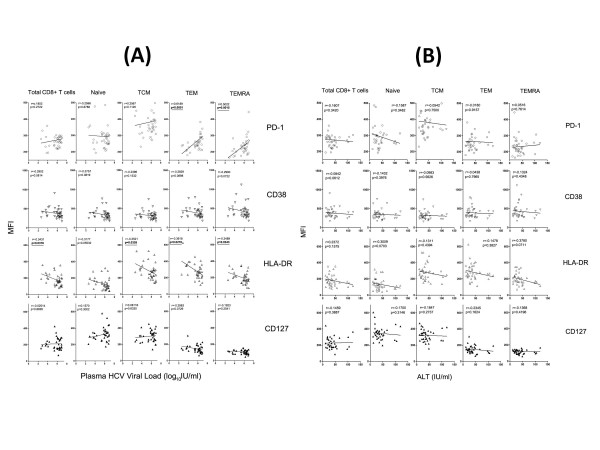
**Correlation analysis of expression of PD-1, CD38, HLA-DR and CD127 on total CD8+ T cell and its subsets with HCV plasma viral load (A) or ALT level (B) in HCV-infected patients**.

Correlation between HCV viral load or ALT and T cell markers was summarized in Figure 4. PD-1 expression on TEM and TEMRA was positively correlated with HCV viral load in HCV infection (TEM: r = 0.6189, p < 0.0001; TEMRA: r = 0.5022, p = 0.0015; Figure 4A). No correlation was found between total CD8+ T cells or naïve/TCM subsets and HCV viral load. In contrast, HLA-DR expression on total CD8+ T cells (r = -0.3431, p = 0.0376), TCM (r = -0.3521, p = 0.0326), TEM (r = -0.3618, p = 0.0278) and TEMRA (r = -0.3489, p = 0.0343) was negatively correlated with HCV viral load in HCV-infected patients (Figure 4A). No correlation between CD38 and CD127 (Figure 4A) expression and HCV viral load appeared in HCV-infected subjects. Unfortunately, we didn't find any correlation between MFI of PD-1, CD38, HLA-DR or CD127 and ALT levels in HCV infected patients (Figure 4B).

### Percentage of CD38+HLA-DR+ cells in HCV chronic infection and its correlation with traditional clinical indicators

**Figure 5:**
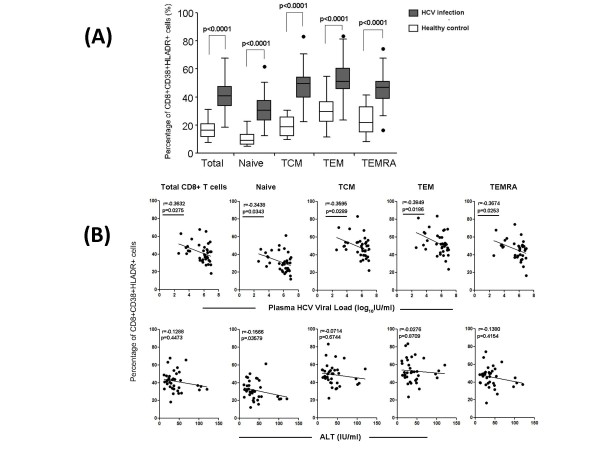
**Percentage of CD38+ HLA-DR+ CD8+ T cells in HCV chronically infected patients and its correlation with traditional clinical indicators**. **(A) **Comparison of percentage of CD38+ HLA-DR+ CD8 T cells on total CD8+ T cell and its subsets in HCV-infected patients (*dark grey boxes*) and healthy controls (*open boxes*). Graphs showed median and interquartile range values of percentages of positive cells. Symbols: ●, outlier values (more than 1.5 times the interquartile range). **(B) **Correlation analysis of percentage of CD38+ HLA-DR+ population on total CD8+ T cell and its subsets with HCV plasma viral load or ALT level in chronic HCV infected patients.

Since several groups[33,35] reported that virus (including HIV and HCV)-specifically activated T cells presenting CD38+ HLA-DR+ (double-positive) phenotype, we also analyzed proportion of CD38+ HLA-DR+ both in total CD8+ T cells and four different subsets. The results showed that percentage of CD38+ HLA-DR+ double positive cells was increased in all four CD8+ T cell subsets as well as total CD8+ T cells compared to healthy controls (p < 0.0001, Figure 5A). In addition, higher percentage of CD38+ HLA-DR+ cells appeared in memory/effector cells than naïve subset both in HCV persistent infected individuals and healthy controls (Figure 5A). Moreover, a significant correlation between percentage of CD8+ CD38+ HLA-DR+ T cells and HCV viral load was found in all CD8+ T cell subsets ((p < 0.05, Figure 5B).

## Discussion

It is widely accepted that HCV-specific CD8+ T cells play an essential role in immune responses to HCV infection and duration and intensity of CD8+ T cell immunity are closely correlated with progression of acute HCV infection [6,36-39]. The maturation and maintenance of CD8+ memory T cells are characterized by their ability to proliferate vigorously and mediate viral elimination upon antigenic re-challenge [40,41]. Memory T cells confer immediate protection in peripheral tissues and mediate recall responses to antigen in secondary lymphoid organs[42]. T cells consist of distinct populations characterized by homing capacity and effector function. Effector memory cells (TEM) migrate to inflammatory peripheral sites and perform immediate effector function[42]. CD8+ TEM expressing CD45RA is defined as TEMRA, which represent the most differentiated type of memory cells and carries the largest amount of perforin and Fas ligand[10,42]. Central memory cells (TCM) usually resident in T cell areas of secondary lymphoid organs and readily proliferate and differentiate into TEM in response to antigenic stimulation though they per se have little or no effector function [15,42]. In this study, we used CD45RA/CD27 instead of CD45RA/CCR7 to divide CD8+ T cell into four different subsets (naïve/TCM/TEM/TEMRA) since several studies have demonstrated that CD45RA+CD27- phenotype (TEMRA) can be found in EBV, HCV, CMV and HIV carriers[43-46]. Our data also showed that more than 90% percent of CD45RA+CD27+ (naïve) CD8+ T cell was consistent with CD45RA+CCR7+ population both in HCV chronic infection and healthy controls (Figure 1C), which further confirm the correspondence of availability of CD27 and CCR7 in the definition of CD8+ T cell subsets. As shown in Figure 1B, HCV-infected patients displayed decreased naïve CD8+ T cells and increased TEMRA subpopulation compared to healthy controls. However, no correlation was found between CD8+ T cell subsets and peripheral HCV viral load or ALT level (data not shown). It is possible that HCV viral persistence would induce more naïve CD8+ T cells to be activated resulting to higher proportion of TEMRA CD8+ T cells emerged. Nevertheless, the activated TEM/TEMRA subsets lose the ability to clear peripheral HCV virus or intraheptic viral replication as seen by HCV persistence.

The activation status of HCV infection was higher than that of healthy controls according to the expression of CD38 and HLA-DR (Figure 2B, 3 and Figure 5). Since the balance between activating and inhibitory marker expression was critical for homeostatic maintenance of CD8+T cell-mediated immune response[21,47-49], we hypothesized that long-term activation of CD8+ T cells might result in enhanced inhibitory marker expression on effector CD8+ T cells. This hypothesis was proved by enhancement of PD-1 and CD38/HLA-DR expression on CD8+ T cells (Figure 3). Interestingly, we found expression of PD-1 on CD8+ TEM (p < 0.0001) and TEMRA (p = 0.0015) were positively correlated with plasma HCV viral load in HCV-infected patients (shown in Figure 4A). Since PD-1/PD-L1 pathway was involved in down-regulating function of activated CD8+ T cells in liver and peripheral circulation[25], it is conceivable that enhancement of PD-1 expression on CD8+ T cells was correlated with capacity of virus replication. Future study of CD8+ T cells resident in liver may probably confirm this correlation and provide more information than current data to assess the role of PD-1/PD-L1 pathway on CD8+ T cell activation, function and HCV viral replication.

Expression level of HLA-DR, but not CD38, on activated CD8+ T cell, including memory and effector subsets, were shown to negatively correlate with circulating HCV viral load (p < 0.05). Unlike HLA-DR, CD38 was considered as a critical marker for T cell activation since it mainly effects in cell adhesion, signal transduction and calcium signaling, but not in antigen presentation process [50]. In accordance with this, this study demonstrated that HLA-DR was a better indicator than CD38 to predict the HCV replication in vivo. Interestingly, the proportion of CD38+ HLA-DR+CD8+ T cells was shown to negatively correlate with HCV viral load both on total CD8+ T cells and its subsets (Figure 5). This result indicated that combination of CD38 and HLA-DR was more accurate to signal the correlation with HCV viral load in chronic HCV infection than employment of CD38 or HLA-DR individually.

CD127 displayed distinguishing characteristics in HCV-infected patients and healthy controls in comparison with expression of PD-1, HLA-DR and CD38. Kaech *et al. *demonstrated that IL-7 receptor (CD127)/IL-7 pathway is critical for maintenance of memory CD8+ T cell homeostasis [28]. In fact, adoptively transferred CD127+CD8+ T cells but not CD127-CD8+ T cells survived in absence of antigen stimulation by homeostatic proliferation via CD127[51]. Therefore, CD127+CD8+ T cells were regarded to have phenotypic and functional features of TCM, while CD127-CD8+ T cells have features of TEM. In this study, CD127 expression on CD8+ T cells decreased when CD8+ T cells differentiated from TCM to TEM, which is consistent with the results reported previously and further indicate that lower expression level of CD127 on TCM and TEM will be closely associated with the impairment of memory CD8+ T cell homeostasis.

Taken together, PD-1, CD38 and HLA-DR expression was increased on all CD8+ T cell subsets while CD127 expression was decreased on TCM and TEM subsets compared to healthy controls. MFI of PD-1 on CD8+ TEM and TEMRA was positively correlated with HCV viral load while HLA-DR molecules on CD8+ T cell subsets (memory/effector) were negatively correlated with HCV viral load in HCV-infected patients. Therefore, PD-1 might be regarded as a simple and easy-manipulated CD8+ T cell marker to assess viral replication in chronic HCV infected patients. These findings will be helpful to understand the interaction between maturation/activation and function of CD8+ T cell and viral replication during HCV long-term persistence.

## Conclusion

PD-1 level on peripheral CD8+ TEM/TEMRA was highly correlated with HCV viral load in chronic HCV-infected patients, which made PD-1 a novel indicator to evaluate the impairment and dysfunction of host CD8+ T cell immunity as well as HCV viral persistence.

## Competing interests

The authors declare that they have no competing interests.

## Authors' contributions

TS, FL and HZ design the study. TS and JZ performed the statistical analysis and interpretation of the data. TS drafted the manuscript. TS, JZ, CX, JL and WZ collected samples and performed benchwork. All authors read and approved the final manuscript.
